# Recent advances in the enzymatic synthesis of lipophilic antioxidant and antimicrobial compounds

**DOI:** 10.1007/s11274-021-03200-5

**Published:** 2021-12-07

**Authors:** Bartłomiej Zieniuk, Ewa Białecka-Florjańczyk, Katarzyna Wierzchowska, Agata Fabiszewska

**Affiliations:** Department of Chemistry, Institute of Food Sciences, Warsaw University of Life Sciences—SGGW, 159c Nowoursynowska St., 02-776 Warsaw, Poland

**Keywords:** Lipase-catalyzed ester synthesis, Lipophilic antioxidants, Food additives, Antimicrobial compounds, Lipase, Lipophilization

## Abstract

Due to the increase in the consumption of highly processed food in developed countries, as well as, a growing number of foodborne diseases, exploration of new food additives is an issue focusing on scientific attention and industrial interest. Functional compounds with lipophilic properties are remarkably desirable due to the high susceptibility to the deterioration of lipid-rich food products. This paper in a comprehensive manner provides the current knowledge about the enzymatic synthesis of lipophilic components that could act as multifunctional food additives. The main goal of enzymatic lipophilization of compounds intentionally added to food is to make these substances soluble in lipids and/or to obtain environmentally friendly surfactants. Moreover, lipase-catalyzed syntheses could result in changes in the antioxidant and antimicrobial activities of phenolic compounds, carbohydrates, amino acids (oligopeptides), and carboxylic acids. The review describes also the implementation of a new trend in green chemistry, where apart from simple and uncomplicated chemical compounds, the modifications of multi-compound mixtures, such as phenolic extracts or essential oils have been carried out.

**Supplementary Information:**

The online version contains supplementary material available at 10.1007/s11274-021-03200-5.

## Introduction

In the last 30 years, the market availability and consumption of processed foods have increased. Based on an analysis of household food distribution in 19 European countries, the median average availability for "ultra-processed" foods was 26.4%, whereas that for processed foods was 19.6%. The median average for processed culinary ingredients was 20.3% and that for unprocessed or minimally processed foods 33.9% (Monteiro et al. 2018). Complex technological processes, combined with a high processing degree of the final product, can have a negative impact on food quality (Zieniuk et al. 2021b). The term "food additive" covers a wide range of ingredient categories with diverse functional characteristics. Overall, six groups may be distinguished: preservatives, texturizing agents, flavourings, colourants, dietary additives and other, miscellaneous agents (Carocho et al. 2014). Food additives are considered key elements to improve the quality and appearance of a foodstuff, along with its safety, throughout the entire life-cycle of a product, from processing, storage and packing to consumption (Sun et al. 2021).

Among the crucial aspects of food product design are durability and safety. Food additives with protective effects can be divided into antioxidants, antimicrobial and anti-browning agents (Carocho et al. 2014). Pursuant to Regulation (EC) No 1333/2008 of the European Parliament and of the Council of 16 December 2008, the category of antioxidants includes substances intended to extend the shelf-life of foodstuffs by protecting them against deterioration as a result of the oxidation process, e.g., fat rancidity and discolouration, whereas protection against food spoilage by saprophytic microorganisms and prevention of pathogen growth are ensured by the addition of preservatives (Carocho et al. 2014).

Nevertheless, the technological application of food additives is a subject of controversy and related to the effects of these substances on human health (Boutillier et al. 2020). One type of food allergy is allergic reactions to food additives. The majority of the population gains tolerance to food antigens; however, if such tolerance is not developed, a specific hypersensitivity reaction occurs (Gultekin and Doguc 2013).

The search for new ingredients is carried out via chemical synthesis, extraction from natural sources or biotechnological production (Cong et al. 2019). Safety reasons and sustainability requirements are currently attracting increased interest in bio-based methods. Furthermore, the preferences of consumers looking for replacements for traditional dietary ingredients are also prompting a broader knowledge of how to obtain food additives that meet the new standards of "naturalness". In other words, the consumers tend to accept the additives synthesized by biotechnological means and accept the compounds which are identical to natural ones or their derivatives. This approach is described as a strong trend in food of the future (Sun et al. 2021). The commercialization of compounds acting as food additives is also facilitated via biocatalysis and processes such as esterification or general enzymatic reactions occurring under milder conditions, with the generation of fewer by-products (Zieniuk et al. 2020, 2021c).

The review summarize the current knowledge about the enzymatic synthesis of lipophilic antioxidants and antimicrobials, particularly dual-functioning compounds. Emphasis was placed on lipase-catalyzed ester synthesis in non-aqueous media. Structural modifications of the molecules, especially those increasing lipophilicity and improving their solubility in lipids, are also discussed. Bioactivity of described compounds, especially antioxidant activity in in vitro assays or as an addition to oils, fats, and emulsions to increase their stability and antimicrobial activity to selected groups of bacteria and fungi are drawn too.

## Biocatalysts – what are they and how do they work?

Enzymes are biocatalysts and regulate the rate at which chemical reactions occur without being altered in the reaction. Biocatalysts are mainly proteins that consist of one or multiple polypeptide chains. Enzyme-assisted reactions are accelerated because a suitable catalyst transforms the substrate into the intermediatory product, the transition state of which is at a lower energy level than that of a non-catalyzed reaction. An enzyme is generally highly specific to both compound to be converted (substrate specificity) and the type of reaction to be catalyzed (reaction specificity) (Belitz et al. 2009; Heckmann and Paradisi 2020). Biocatalysis is defined as the use of natural substances that include enzymes from biological sources or whole cells to speed up chemical reactions. Both approaches can be applied at laboratory and industrial levels. Most enzyme properties can only be revealed with the use of purified enzymes, but the removal of protein impurities is an expensive stepwise process (Belitz et al. 2009; Wang et al. 2019).

Enzymes can be obtained from plants, animals and microorganisms. Although some commercial catalytic proteins have been derived from plant sources or extracted from animal tissues, microorganisms, such as fungi, bacteria or yeasts, are a preferred source of industrial biocatalysts (Watson and Soumatainen 1984; Heckmann and Paradisi 2020).

The enzyme-based synthesis of chemicals can occur at moderate pH and temperature conditions and low pressure (Matsumoto et al. 2019). As biocatalysts are attractive for use in synthetic applications, especially in regio- and stereoselective reactions (Heckmann and Paradisi 2020), it is not surprising that the number of biocatalytic processes in the industry has increased rapidly from 60 in 1990 to several hundred in 2019 (Heckmann and Paradisi 2020). Nowadays, enzymes are used in the biofuel industry, food and feed industries, the detergent and paper industry, the pharma industry, chemical synthesis, diagnostics, the textile industry and bioremediation.

They can also replace traditional catalysts based on toxic or scarce metals, which is an important aspect in environmentally friendly manufacturing processes (Poliakof and License 2007). The key for the success of industrial applications of enzymes was the discovery of the possibility to immobilize proteins with retention of their function and enhancing their stability. This allowed biocatalysts to be recycled, reducing the costs by lowering the quantity of enzyme that has to be isolated. Enzyme immobilization may be mechanical or physiochemical (divided into covalent or adsorption immobilization). The second milestone in enzyme commercialization was the synthesis of recombinant proteins. Molecular biology, directed evolution, statistical tools, rational design and system biology revolutionized biocatalysis and overcame some major problems regarding solvent tolerance and extended substrate scope (Heckmann and Paradisi 2020).

Nevertheless, there are still some issues, including the prolonged development time and difficulties in down-stream processing, along with the dependence on expensive cofactors. In this context, interdisciplinary teams are planning the most suitable chemical routes for integrated enzyme engineering and process development, new concepts for bioreactor engineering are being expanded, and efficient recycling systems are available for NADH, NADPH and ATP (Wu et al. 2021).

## The state of the art in the lipase-catalyzed modification of food products

The traditional use of enzymes in food processing refers mainly to the modification and breakdown of biomaterials (Raveendran et al. 2018). Most of these enzymes belong to the class of hydrolases, such as amylases (used in bread making and in the manufacture of corn syrups), invertases (involved in the hydrolysis of sucrose), pectinolytic enzymes (breaking down polysaccharides found in plant cell walls), proteases (hydrolysing of peptide bonds of proteins) and lipases, which hydrolyze ester linkages in glycerides. Of these, the latter are the most commonly used enzymes not only in the food industry (Jaeger and Eggert 2002) but also in chemical syntheses. Details information about the use of enzymes, and especially lipases can be found in the review articles of Belitz et al. (2009), Chandra et al. (2020), Coelho and Orlandelli (2021), Heckmann and Paradisi (2020), Mehta et al. (2021), and Raveendran et al. (2018).

Esters bonds can be hydrolyzed by both esterases and lipases, of which the former are referred to as 'true' esterases (EC 3.1.1.1) and the latter as triacylglycerol hydrolases (EC 3.1.1.3)); they can be distinguished by the reactions they catalyse. Esterases hydrolyze "simple" short-chain esters, and lipases break down water-insoluble triacylglycerols (Lopes et al. 2011). Due to the ability of some lipases to hydrolyze of short-chain esters, other criteria have been proposed. First, the interfacial activation phenomenon, which has not been observed in esterases, as well as the presence of a lid domain have been used for many years to distinguish these groups of enzymes. Unfortunately, this criterion is also not ideal as not all lipases meet these conditions (Bracco et al. 2020). Another way to differentiate these enzymes is based on their activity in organic solvents and the capability of acting in low-water activity environments, where only lipases and cutinases (closely related enzymes) are active (Bracco et al. 2020).

Although in natural environments, lipases are responsible for the hydrolysis of lipids to glycerol and fatty acids, in the absence of water these enzymes can catalyze the reverse reactions such as esterification, interesterification transesterification and the transfer of acyl groups from esters to other nucleophiles (e.g., amines and thiols). Besides, triacylglycerols lipases can also catalyze the formation of other esters because of their relatively high substrate tolerance. They are highly resistant to unfavourable temperature and pH levels and to different organic solvents; an additional advantage is that they do not require the presence of a cofactor. All these features allow them to be used in chemical syntheses and in combination with the previously discussed advantages of enzymes determines their great importance (Coelho and Orlandelli 2021; Jaeger and Eggert 2002).

The natural substrates of lipases are triacylglycerols (TAGs), and in aqueous media, TAGs are hydrolysed to free fatty acids and glycerol, monoacylglycerols or diacylglycerols, depending on the regioselectivity of the enzyme. Catalyzing the hydrolysis, esterification and interesterification of acylglycerol lipases allow modifying the properties of these lipids by altering the location of fatty acid chains in the glyceride and replacing them with new ones. Such tailored triacylglycerols with modified physicochemical properties are nutritionally important and have a large potential, for example as human milk fat or cocoa butter substitutes, low-calorie triacylglycerols and oils enriched with specific fatty acids (Chandra et al. 2020; Mehta et al. 2021).

Because of their previously mentioned substrate acceptance and stability in many organic solvents, lipases catalyze the biotransformation of various compounds containing a carboxyl group, such as esterification, transesterification and aminolysis in which the typical nucleophile (water) is replaced by alcohol or an amine. A long-known example of the use of lipases is the synthesis of aromatic food additives, which often have esters or lactone structures and are applied not only in the food sector but also in the cosmetic industry. The production of low-molecular weight esters, such as flavour compounds by means of biocatalysis is a useful and promising alternative green tool which offers high yields in mild reaction conditions (Jaiswal and Rathod 2020). For example, the enzymatic syntheses of short-chain fatty acids esters with naturally available terpene alcohols as well as hexyl esters (green note flavour compounds) have been described (Sa et al. 2017).

Apart from the use of lipases for the synthesis of food additives (both known and new), these enzymes are a valuable tool in the modification of food additives with respect to the ester or carboxyl groups. The objective of such modifications, as a rule, is to change or improve the organoleptic properties of the ingredients as well as their miscibility with lipids as the consequence of hydrophile-lipophile balance (HLB) (Białecka-Florjańczyk et al. 2018). In the latter case, the change is achieved via introducing an element characterized by a distinct hydrophilic or lipophilic character into the ester molecule by means of a lipase-catalyzed reaction. Examples of such modifications applied to different groups of food products will be presented below.

**Fig. 1:**
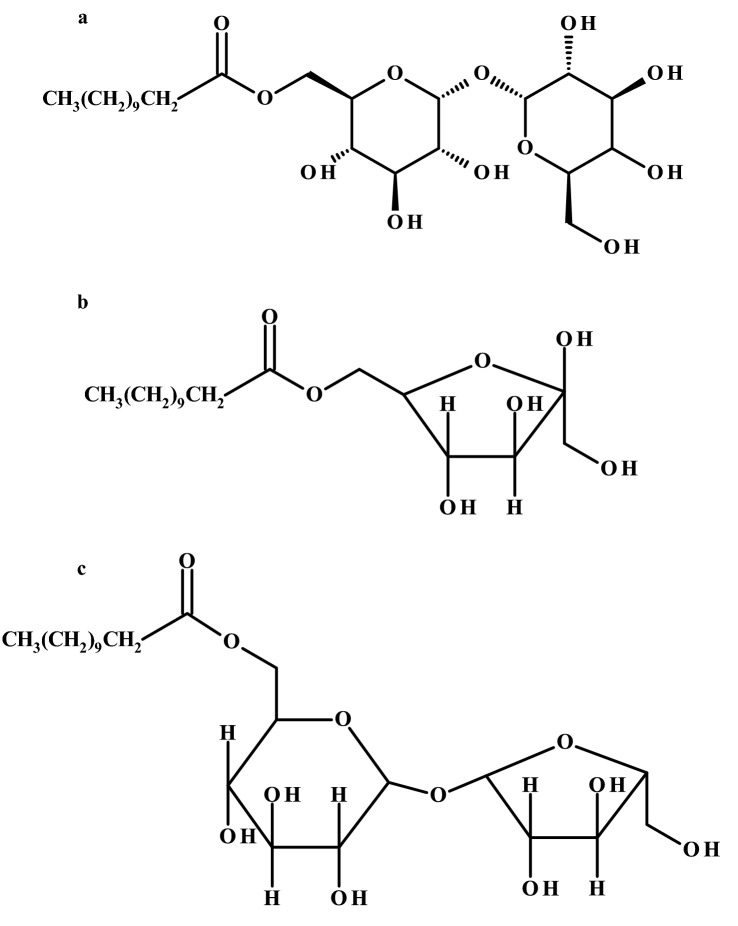
Examples of sugar esters obtained in lipase-catalyzed reactions: **a** trehalose laurate, **b** fructose laurate, **c** sucrose monolaurate

Esters of mono and disaccharides as well as fatty acids are nonionic surfactants widely exploited in the food and cosmetics industries, as well as in the oral care and medical supply fields. They can be synthesized by enzymatic reactions in which sugars are acylated on their primary hydroxyl groups using different lipases as catalysts. Most of the lipases applied in ester synthesis in nonaqueous media appear to be of microbial origin (e.g. *Candida antarctica, C. rugosa, C. cylindracea* or *Rhizomucor miehei*). However, problems may arise when choosing the appropriate solvent because the solubilities of sugars and fatty acids are generally markedly different. Acetonitrile, *tert*-butanol and ethyl methyl ketone are good solvents, but many lipophilization reactions are performed in solvent-free systems or with the use of ionic liquids and deep eutectic solvents (Neta et al. 2015). Depending on the esterification degree and the nature of the fatty acid and/or sugar, a range of sugar esters can be synthesized (Fig. 1a). Differing in their surface activity and emulsifying capacity, they are promising for applications in the food industry.

The use of the native forms of polysaccharides for food applications is not always possible due to solubility problems, but the presence of reactive groups (hydroxyl and carboxyl, acetamido or amino groups) enables the change of hydrophilic/hydrophobic balance via their functionalization due to the change in the length of the alkyl (fatty acid) residue. Such amphiphilic polysaccharides can act as polymeric non-ionic surfactants while keeping some attributes of the starting materials e.g. emulsifying, gelling and film-forming properties. In some cases, as a result of such transformation, the polysaccharide may gain a favourable change in its characteristics. Therefore, the reaction of starch with various acylating agents has been performed, leading to a more hydrophobic thermoplastic polymer and extending its use as a carrier for targeted drug delivery systems and in biomedical applications. Hydrophobicity, viscosity and emulsifying properties have significantly been improved because of the enzymatic esterification of starch with rosin acid (Karaki et al. 2016).

**Fig. 2:**
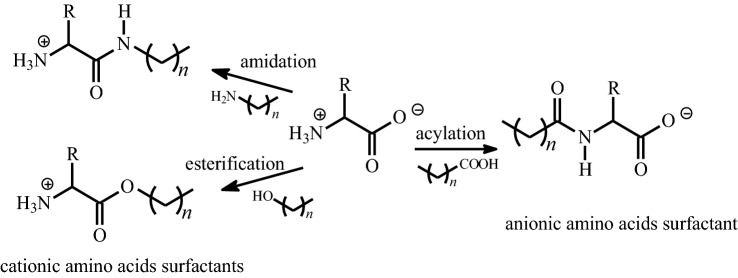
The possibilities of lipase-catalyzed functionalization of the amino acids

Amino acids (or oligopeptides) comprise another group of natural compounds susceptible to various modifications. Having at least two distinct functional groups, they can be easily functionalized, and the hydrophobic chain can be introduced into the amino acid structure to create lipoaminoacid (lipopeptide), a new group of bio-sourced surfactants. The introduction of the hydrophobic chain at the amino group by acylation with a fatty acid leads to anionic *N*-acyl amino acids surfactants (AAS), whereas condensation of the carboxyl group of the amino acid with fatty alcohols or fatty amines produces cationic alkyl ester and alkyl amide AAS, respectively (Fig. 2). Additionally, cationic surfactants based on amino acids show excellent antimicrobial and antifungal properties. Structurally, these compounds can be considered as analogues of native lipopeptides since they are cationic amphiphiles consisting of amino acids linked to a hydrophobic moiety. Amino acid-based surfactants are characterized not only by high biodegradability and low toxicity but also are environmentally friendly and have a high water tolerance (Pinazo et al. 2016; Tripathy et al. 2018).

Lipophilization also has a significant impact on the action of antioxidant food additives. Most natural antioxidants are polyhydroxy compounds, such as flavonoids or phenolic acid derivatives, and exhibit hydrophilicity. This structure, limiting oil solubility, generally restricts their applications under hydrophobic conditions. Lipophilization involves the reaction with fatty acids in the case of flavonoids or with lipophilic alcohols in the case of phenolic acids (Figueroa-Espinoza and Villeneuve 2005). The enzymatic synthesis of lipophilic antioxidants (Lipo-PCs—lipophilic phenolic compounds) is a significant issue nowadays due to both consumer preferences and a limited number of compounds of this type of natural origin. Although lipophilization of phenolics is supposed to improve oil solubility, it may also impart novel properties to the molecule, especially bioavailability and the biological properties of the starting compounds may be improved (Liu et al. 2014).

**Fig. 3:**
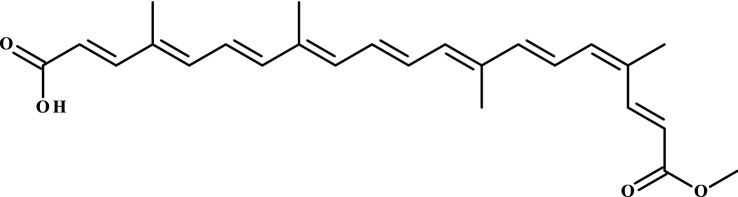
Chemical structure of bixin

Finally, it is worth mentioning that in some cases, enzymatic hydrophilization is applied to make the compound more suitable for water-based food formulations. Such a procedure takes place in the case of bixin (Fig. 3), an apocarotenoid of natural origin used as colourant in the food industry. Bixin apart from one carboxylic acid and one methyl ester group, contains a polyene chain which determines its hydrophobic properties. Water solubility can be increased by enzymatic transesterification with a hydrophilic substance such as sorbitol or L-ascorbic acid but in the latter case, the main goal of the authors was to inhibit the oxidative degradation of the dye (Humeau et al. 2000).

It should be emphasized that the main goal of enzymatic lipophilization of food components is to make these substances soluble in lipids and/or to obtain an environmentally friendly surfactant. However, at the same time, such a change in the structure of the compound may have a significant impact on its other properties, in particular biological activity. Therefore lipophilization can be considered as the synthesis of multifunctional food additives.

## Application of biocatalysis in the synthesis of lipophilic antioxidants and antimicrobial compounds

This section provides comprehensive information about recent advances in the enzymatic synthesis of lipophilic antioxidants and antimicrobial agents and their various applications. Emphasis is placed on derivatives of phenolic compounds as well as ascorbic and erythorbic acids, carbohydrates, sugar alcohols and certain carboxylic acids and their biological activities. The original research articles used for this section were published within 2015–2021. Additionally, Table 1 compiles the patented processes that use lipases for molecule modifications. By the end of October 2021, 15,082 results containing the word "lipase" were found in the PATENTSCOPE database of the World Intellectual Property Organization (WIPO). Out of 130 patents that appeared in 2021, 35 are listed in Table 1. Lipases are mainly used for biodiesel synthesis, triacylglycerols modifications or the kinetic resolution of enantiomers.

**Table 1:** The patented processes in 2021 that use lipases for molecule modifications ( source: PATENTSCOPE database of World Intellectual Property Organization, WIPO)

Patent number	Title	Lipase origin	Additional information	Patent office
US332611592	Enzymatic Enrichment Of N-3 Fatty Acids In The Form Of Glycerides	Lipases of different origins	–	USA
CN326499989	Immobilization method of liquid lipase and preparation method of sucrose-6-acetate	*Thermomyces lanuginosus* or *Rhizomucor miehei*	–	China
CN326441279	Preparation method of grease rich in OPL and OPO and product thereof	Immobilized lipase from *C. antarctica*	Enzymatic synthesis of triglycerides rich in oleic and linoleic acids	China
CN326508917	Immobilized lipase catalyzed citric acid functionalized beta-cyclodextrin and preparation method thereof	Immobilized lipase B from *C. antarctica*	–	China
CN327302069	Preparation method of soybean oil-based oleogel	*T. lanuginosus, R. miehei* or *C. antarctica*	Enzymatic alcoholysis reaction to obtain soybean oil-based 2-monoglycerides used to preparation of oleogels	China
CN324357875	Special grease base oil for functional food as well as preparation method and application of special grease base oil	*Staphylococcus caprae*	Enzymatic transesterification of different oils to improve their health benefits	China
US329767749	Production of Fatty Acid Estolides	*Candida rugosa*	–	USA
CN327303149	Method for preparing D-*p*-methylsulfonyl phenyl serine ethyl ester through immobilized enzyme catalysis	*Candida rugosa*	–	China
CN321747108	Synthetic method of brazilin natural product ( +)-Brazilin	*Burkholderia cepacia*	Lipase-catalyzed asymmetric reaction as a part of the process	China
NZ318608852	Method for lowering iodine value of glyceride	*Rhizopus oryzae*	Lipase-catalyzed glycerides esterification or transesterification with saturated fatty acids for lowering iodine value	New Zealand
CN327305950	Method for recovering high-content natural d-alpha-tocopherol succinate from leftovers	No data	Lipase is used to hydrolyze the methyl ester to obtain d-alpha-tocopherol succinate	China
WO2021196881	Triglyceride-type polyunsaturated fatty acid, preparation method therefor and application thereof	Lipases of different origins	Lipase-catalyzed process	WIPO
IN334864549	Chemo-enzymatic process for synthesis of molnupiravir	*C. antarctica* lipase B	Lipase-catalyzed esterification as a part of the process	India
CN328301391	Phenolic acid starch ester as well as preparation method and application thereof	*C. antarctica*	–	China
CN323903949	Application of jasmine root fermentation extract in preparation of anti-inflammatory cosmetics or drugs	*C. antarctica*	Utilization of lipase to obtain jasmine root extract	China
CN326422375	Method for preparing partial glyceride through glycerolysis reaction	*Streptomyces* sp.	–	China
CN328292254	Lipase and application thereof in hydrolysis of astaxanthin ester	*Aspergillus fumigatus*	–	China
CN328302211	Synthesis method of enzyme-catalyzed poly(1,4-butanediol carbonate)	*C. rugosa* or *C. antarctica*	–	China
CN328293762	Edible vegetable oil with low content of 3-chloropropanol ester as well as preparation method and application of edible vegetable oil	*C. antarctica*	Lipase used for reducing the content of monoglyceride and diglyceride in the oil	China
CN329006531	Biocatalyst utilizing two-dimensional polyamide to immobilize lipase and method for preparing biodiesel by catalyzing soybean oil	*C. antarctica* lipase B	–	China
CN328271449	Beef tallow substitute fat as well as preparation method and application thereof in hotpot condiment	*T. lanuginosus, R. miehei* or *C. antarctica*	Lipase-catalyzed transesterification of lipids	China
WO2021182501	Method for producing fat/oil	Lipases of different origins	Lipase-catalyzed modification of lipids	WIPO
CN327322690	Method for catalytically synthesizing sucrose fatty acid ester by lipase in organic solvent	*C. antarctica*	–	China
CN330938399	Method for catalyzing hydrolysis of organic ester by high internal phase emulsion	*C. rugosa, C. antarctica, B. cepacia*	–	China
CN330060811	Production process and application of (2S,5S)-2,5-hexanediol	No data	Lipase-catalyzed asymmetric reaction as a part of the process	China
CN330941090	Immobilized enzyme catalyst, preparation method thereof and application of immobilized enzyme catalyst in synthesis of vitamin A palmitate	*Candida* sp. 99–125	–	China
CN328274550	Method for catalytically synthesizing sucrose ester by using amorphous sucrose	*T. lanuginosus* or *C. antarctica*	–	China
CN330063778	Non-aqueous-phase enzymatic synthesis method of low-molecular-weight 6-O-PGA-L-ascorbic acid	Lipases of different origins	–	China
CN327324605	Method for extracting alpha vitamin E from deodorized distillate	No data	Lipase-catalyzed esterification as a part of the process	China
CN328275546	Method for extracting soybean vitamin E from non-soybean deodorized distillate	No data	Lipase-catalyzed esterification as a part of the process	China
WO2021201210	Method for modifying oil and fat containing food	Lipases of different origins	–	WIPO
WO2021204747	Method for manufacturing *sn-2* palmitic triacylglycerols	*T. lanuginosus*	Lipase-catalyzed synthesis of 1,3-dioleate-2-palmitate-glycerol	WIPO
CN330943213	Method for removing free fatty acid in grease by enzyme catalysis	Lipases of different origins	–	China
CN329976346	Method for preparing hydrocarbon fuel from waste cooking oil	No data	Lipase used for hydrolysis of waste cooking oil	China
CN328989807	Method for concentrating DHA (docosahexaenoic acid) in *Schizochytrium limacinum* grease	*A. oryzae* or *C. antarctica*	Lipase used for hydrolysis of *S. limacinum* lipids	China

### Enzymatic modification of phenolic compounds

Phenolic compounds, are secondary metabolites with various chemical structures that are readily used for enzymatic modifications. The common part of these substances is the presence of an aromatic ring with a hydroxyl substituent. Despite the many biological activities of phenolic compounds previously described in review articles (Durazzo et al. 2019; Lima et al. 2019), phenolics are often poorly soluble in both water and fat, which can be overcome by enzymatic esterification. Esters of ferulic acid are prominently the objects of studies on the enzymatic synthesis of compounds with high biological activity.

**Fig. 4:**
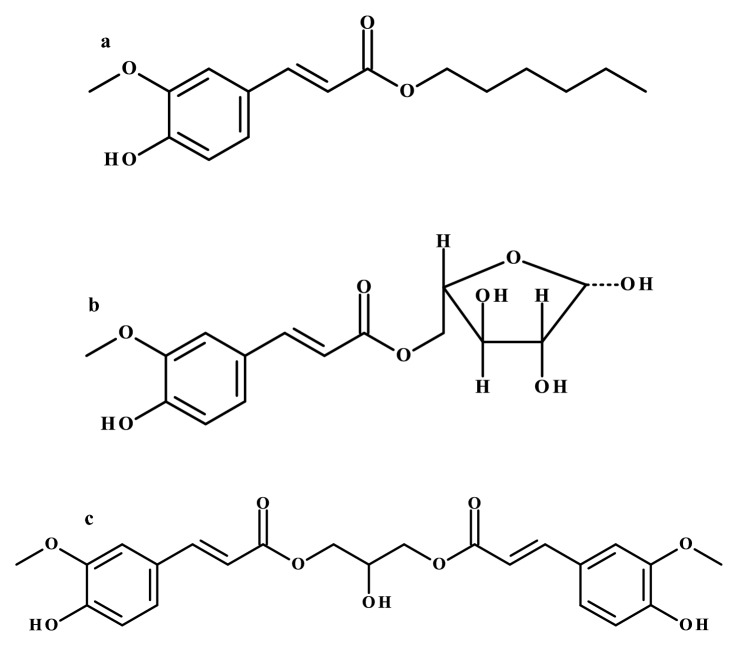
Chemical structures of enzymatically obtained ferulic acid derivatives: **a** hexyl ferulate, **b** L-arabinose ferulate, and **c** 1,3-diferuloyl-*sn*-glycerol

For instance, alkyl ferulates synthesized by esterification of the ferulic acid with alcohols of 4–12 carbon atoms in a molecule catalyzed by lipase B from *C. antarctica* were the subject of studies by Shi et al. (2018, 2019). Among the obtained compounds, hexyl ferulate (Fig. 4a) remarkably inhibited the growth of *E. coli* and *L. monocytogenes*. It was shown that this ester was able to lyse cells, disrupt cell membranes and affect the protein expression system, causing changes in the conformation and content of membrane proteins. The described ester has also been used as a dual-function additive with antioxidant and antimicrobial properties to American sturgeon caviar, which is rich in polyunsaturated fatty acids and, like other fish or seafood products, is susceptible to contamination with *L. monocytogenes*. Hexyl ferulate was able to limit bacterial growth for 7 days and, compared to the negative control (without ester) the difference in bacterial number was more than 7 log cycles (Shi et al. 2019). Table S1 provides a summary of the antimicrobial activities of enzymatically obtained esters.

Antonopoulou et al. (2018) obtained, however, contrasting results, using hydrophilization instead of lipophilization. Five different feruloyl esterases from *Myceliophthora thermophila* were used as biocatalysts in the reaction of vinyl ferulate with L-arabinose. The detergent-less microemulsion consisting *n*-hexane:*tert*-butanol:100 mM MOPS-NaOH (pH 8.0) with the ratio of 19.8:74.7:5.5 (v/v/v) was the optimized reaction medium, and using the FaeA1 esterase allowed obtaining the highest transesterification yield after 8 h at 50 °C. Synthesized L-arabinose ferulate (Fig. 4b) was capable of scavenging DPPH radicals and was not toxic to human skin fibroblasts at a concentration of 1 mM.

Over the last few years, other phenolic acids have also gained interest in enzymatic modification to improve their biological activity. Gholivand et al. (2017) synthesized methyl, hexyl, dodecyl and octadecyl esters of dihydrocaffeic acid, a saturated derivative of caffeic acid in different ionic liquids, with Novozym 435 as a biocatalyst. Elongation of the alkyl chain resulted in a decrease in the scavenging activity in the DPPH assay, and the opposite results were achieved in the β-carotene bleaching test. Zieniuk et al. (2020, 2021a, 2021c) studied the synthesis, antioxidant and antimicrobial properties of different esters of phenolic compounds—phenylacetic and phenylpropanoic acids, their derivatives and analogues. Besides using CALB, *Yarrowia lipolytica* biomass was used as biocatalyst. The resulting esters retained their antioxidant activity, and the authors proved that esters of 3-(4-hydroxyphenyl)propanoic acid showed antibacterial activity against *L. monocytogenes* PCM 2191. The MIC (minimum inhibitory concentration) and MBC (minimum bactericidal concentration) values decreased with increasing the alkyl chain length of the ester, and octyl 3-(4-hydroxyphenyl)propanoate was the most active ester. Thus, lipophilization of phenolic acid through its esterification with linear alcohols allowed to obtain more active compounds compared to their precursor (Zieniuk et al. 2021a).

Another possibility of lipophilization of phenolic compounds is the synthesis of structured triacylglycerols, so-called phenolipids. Using this approach, it is possible to obtain acylglycerols with antioxidant properties, strongly absorbing ultraviolet (UV) radiation and with an an emulsifying effect; they could be used as moisturizers in anti-wrinkle cosmetics (Compton et al. 2018). Transesterification of ethyl ferulate with soybean oil with a high diacylglycerol content in the presence of Novozym 435 yielded several reaction products, of which 1,3-diferuloyl-*sn*-glycerol (Fig. 4c) and 1-feruloyl-*sn*-glycerol were the main products. The compounds after separation by flash column chromatography were subjected to UV absorption assays and could be suitable substitutes for commercial UVB absorbers and UVA-II absorption enhancers when combined with UVA- and UVB-absorbing compounds (Compton et al. 2020).

Rychlicka and Gliszczyńska (2020) used lipase B from *C. antarctica* to modify *p*-methoxycinnamic acid, whose anticancer, antidiabetic, neuro- and hepatoprotective activities are well documented in the literature. To overcome its low bioavailability, the authors proposed and optimized enzymatic interesterification of egg-yolk phosphatidylcholine with ethyl *p*-methoxycinnamate.

In the past few years, lipophilic esters of vanillyl alcohol, tyrosol and hydroxytyrosol have attracted considerable interest. For example, vanillyl alcohol with confirmed antioxidant properties but low solubility in lipids and organic solvents was esterified with e.g. hexanoic and ricinoleic acids or menhaden oil. The combination of this phenolic alcohol and the above-mentioned acyl donors resulted in improved solubility and antioxidant activity in lipids and ameliorated oil oxidative stability, making them also better antimicrobial agents compared to their precursors (Zieniuk et al. 2021b; Park et al. 2020; Natalia et al. 2016). Similarly, several lipophilic tyrosyl and hydroxytyrosyl esters were synthesized via lipase-catalyzed reactions and exhibited antioxidant activities (Zhou et al. 2017). For example, hydroxytyrosyl eicosapentaenoate was an effective agent in the stabilization of fish oil, fish oil-in-water emulsions and microencapsulated fish oil (Akanbi and Barrow 2018).

Kojic acid (5-hydroxy-2-(hydroxymethyl)pyran-4-one) is a secondary metabolite of some fungi of the genus *Aspergillus* and commercially used as a whitening agent with antioxidant activity in the cosmetics industry. Lajis et al. (2017) synthesized 7-*O*-kojic acid monopalmitate which exhibited better depigmenting and antioxidant activities than kojic acid. The authors suggested that the obtained ester had an enhanced chemical stability, and in assays with *Danio rerio* embryos, it showed higher hydrophobicity compared to kojic acid and increased cell membrane permeability, which resulted in reduced tyrosinase activity and melanin formation inhibition (Lajis et al. 2017). Ishak et al. (2018) optimized the enzymatic synthesis of kojic and palmitic acids, and the obtained derivative with pseudoplastic behaviour can potentially be applied in the formulation of lotions.

Scientists dealing with enzymatic synthesis are also interested in epigallocatechin-3-*O*-gallate (EGCG), a major constituent of green tea catechins with proven biological activities. In recent years, different proposals of EGCG modification have been presented. Zhu et al. (2017) performed catechin lipophilization with vinyl acetate via Lipozyme TL IM (*T. lanuginosus* lipase immobilized on a silica gel). Three acetylated derivatives were identified and were better antioxidant agents in soybean oil than EGCG (Zhu et al. 2017). In the study of Nitta and Iwamoto (2019), lipase-catalyzed polymerization of EGCG, divinyl adipate and sugar alcohols was investigated. The obtained poly(EGCG adipate-*co*-sugar alcohol-adipate)s have potential application in the cosmetics industry, because of the gradual release of EGCG, its antioxidant activity and non-toxicity to human neuroblastoma cells.

A new approach to obtaining lipophilic compounds is the enzymatic modification of plant extracts rich in polyphenols, and the following extracts have already been modified: bamboo-leaf extract, which consists mainly of orientin, isoorientin, vitexin and isovitexin (Ma et al. 2015) grape seed proanthocyanidins (Chen and Yu 2017) and anthocyanins from alpine bearberry (Yang et al. 2018). Laurate esters of bamboo leaf flavonoids significantly reduced the content of acrylamide in fried potato crisps. Acylated flavonoids in the concentrations of 0.05 and 0.1% lowered the amount of this hazardous compound by 44.5 and 46.9%, respectively. The authors suggested that esters of bamboo leaves flavonoids scavenged reactive carbonyls formed in Maillard reactions, which are responsible for the formation of acrylamide in food (Ma et al. 2015). Chen and Yu (2017) obtained mono-, di- and tri-lauroylated derivatives of grape seed proanthocyanidin components, such as epigallocatechin, catechin, epicatechin or epicatechin gallate. The radical scavenging activities of lipophilic derivatives of these flavan-3-ols suggested their potential wide application in the food industry. Moreover, Yang et al. (2018) synthesized a novel compound, cyanidin-3-*O*-(6ʹʹ-dodecanoyl)galactoside with the use of anthocyanin isolated from alpine bearberry (*Arctostaphylos alpine* L.) and lauric acid in the presence of Novozyme 435; the introduction of saturated fatty acids to the structure of polyphenol compound markedly improved ester lipophilicity and thermostability while maintaining similar antioxidant activity (Yang et al. 2018).

### Lipase-catalyzed synthesis of esters of ascorbic and erythorbic acids

Fatty acid ascorbyl esters are well-known food additives exhibiting antioxidant properties and are listed as substances permitted in the European Union with E 304(i) and E 304(ii) numbers for ascorbyl palmitate and ascorbyl stearate, respectively. The enzymatic synthesis of ascorbic acid esters and their effect on the stability of oils and emulsions are well known, and in the last years, mainly the modifications of reaction conditions have been studied (Li et al. 2018; Stojanović 2015). An outstandingly interesting paper was presented by Bhatia et al. (2019). In fully biotechnological processes the authors synthesized copolymer of poly(3-hydroxybutyrate-*co*-3-hydroxyvalerate) (PHBV) functionalized with ascorbic acid using, CALB-catalyzed esterification, and the polymeric substrate was produced by engineered *E. coli* YJ101. The obtained copolymer was characterized by a lower degree of crystallinity as well as a higher thermal stability and hydrophilicity in comparison with its precursor. Furthermore, functionalized biomaterial exhibited antioxidant activity and was more biodegradable, which can increase its medical applications. Although PHBV and its derivatives cannot be used in food-related applications, the authors showed a certain direction of research in which polymers can be enzymatically modified to obtain valued chemical compounds.

**Fig. 5:**
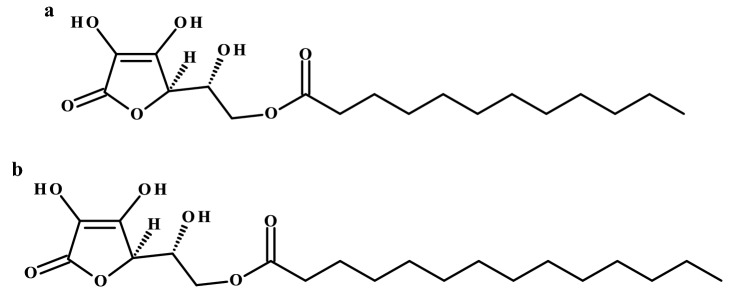
Chemical structures of **a** erythorbyl laurate and **b** erythorbyl myristate

In recent years, the stereoisomer of ascorbic acid, erythorbic acid has been enjoying greater popularity. Since 2017, a team of scientists from the Republic of Korea have been publishing a series of original articles confirming the antimicrobial, antioxidant and anti-inflammatory properties of erythorbyl laurate (Fig. 5a) and erythorbyl myristate (Fig. 5b) obtained in reactions catalyzed by Novozym 435. Erythorbyl laurate has been presented as a multi-functional food additive. The obtained lauric ester of erythorbic acid as a non-polar compound was able to impede the development of peroxides in soybean oil emulsions and showed surfactant properties (Park et al. 2017). Apart from its antioxidant activity, its antibacterial properties have been evaluated against Gram-positive foodborne pathogens and bacteria such as *L. monocytogenes, S. aureus*, and *B. cereus* were susceptible to the action of described ester. The mechanism of bactericidal activity probably depends on changes in the integrity and permeability of cell membranes (Park et al. 2018). Erythorbyl myristate showed similar properties (Park et al. 2021). The research on the antibacterial activity of erythorbyl laurate has also been extended to transcriptomic analysis of *S. aureus* under stress conditions, and this ester has been proposed as cell wall-active compound; RNA-Seq analysis revealed that genes related to cell growth were down-regulated and that cell wall stress stimulation genes were up-regulated (Park et al. 2019). The authors also evaluated the anti-inflammatory effect of erythorbyl laurate and could show that it suppressed TNF-α-induced adhesion of monocytes to the vascular endothelium, making it a promising functional additive in the prevention of vascular inflammation (Ha et al. 2021).

### Enzymatic synthesis of sugar esters

Sugar esters are other, valuable compounds that may result from lipase-catalyzed esterification. They are composed of different sugars and fatty acids and can potentially be used as antimicrobial agents or emulsifiers due to their amphiphilic nature. Lee and Kim (2016) synthesized fructose monolaurate (Fig. 1b) by transesterification of fructose with methyl laurate, using CALB as a biocatalyst and *tert*-butanol containing 20% dimethyl sulfoxide as a solvent mixture. The aforementioned ester was able to suppress the growth of *Streptococcus mutans*, bacteria responsible for dental decay, and other food spoilage microorganisms, e.g., *B. coagulans*, and *Geobacillus stearothermophilus* (Lee and Kim 2016). Shao et al. (2018) obtained a similar compound in the reaction of sucrose and vinyl laurate catalyzed by Lipozyme TL IM in 3CIM(EO)][NTf_2_] ionic liquid. The ester was evaluated by antibacterial activity against four bacteria including *L. monocytogenes*, *B. subtilis*, *S. aureus*, and *E. coli*. The values of MIC and MBC were determined, and the time-kill assay was applied to assess the antibacterial properties. Sucrose monolaurate (Fig. 1c) showed a higher activity against Gram-positive bacteria compared to *E. coli*. The authors proposed a mechanism of ester action that was related to the damage of the cell membrane integrity (Shao et al. 2018).

Another example of sugar ester enzymatic synthesis was provided by Ning et al. (2017), who obtained neokestose laurate in a CALB-catalyzed reaction. Neokestose is a fructooligosaccharide with prebiotic and other attractive properties. Similar to the abovementioned papers, a mixture of solvent (20% DMSO in 2-methyl-2-butanol) was used as reaction medium, and the synthesized ester was defined by the authors as a dual functional agent with antibacterial and emulsification activities (Ning et al. 2017).

Unusual and fascinating methods of obtaining glycolipids were presented by Siebenhaller et al. (2018) and El-Baz et al. (2021). In the first article honey and agave syrup were used simultaneously as solvents and substrates for the enzymatic transesterification of four fatty acid vinyl esters (vinyl octanoate, vinyl decanoate, vinyl laurate, and vinyl palmitate). Due to the low water content and liquid form reminding sugar-based deep eutectic solvents (DES), these substrates were successfully used in the synthesis of glycolipids with potential use in cosmetics and pharmaceutical industries (Siebenhaller et al. 2018). El-Baz et al. (2021) proposed single-cell oils (SCO) generated by *Cunninghamella echinulata*, *Umbelopsis isabellina* or *Nannochloropsis gaditana*, as well as olive oil and eicosapentaenoic acid (EPA) concentrate, as acyl donors in the enzymatic synthesis of glucose fatty acid esters. Glucose esters with higher contents of polyunsaturated fatty acids were more effective against pathogenic bacteria. The *C. echinulata* oil-glucose esters also exhibited strong insecticidal activity, and all synthesized esters induced apoptosis of the SKOV-3 ovarian cancer cell line.

### Enzymatic modification of other valuable compounds

**Fig. 6:**
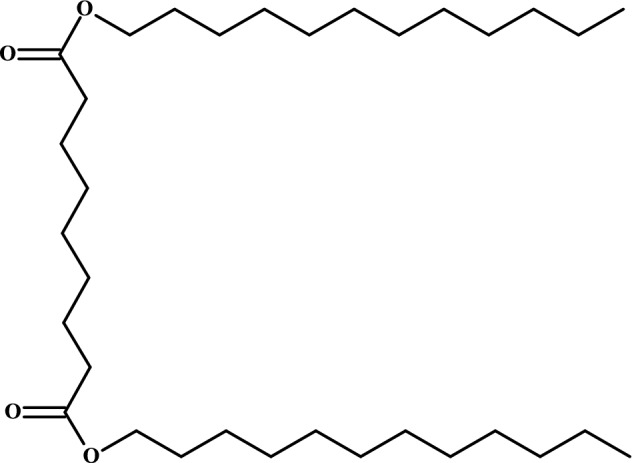
Chemical structure of dilauryl azelate

Other important compounds are azelaic acid derivatives. Azelaic acid, which occurs in natural environments, is a dicarboxylic acid that is effective in the treatment of acne. However, because of its low solubility, crystalline form and high melting point, its usage in cosmetics and pharmaceutical industries is limited. Dilauryl azelate ester (Fig. 6) was successfully synthesized with Novozym 435 by applying the response surface methodology. In comparison with azelaic acid, its dilauryl ester was non-toxic to 3T3 normal fibroblast cells and had a comparable antibacterial activity against *S. epidermidis* S273 (Khairudin et al. 2018).

**Fig. 7:**
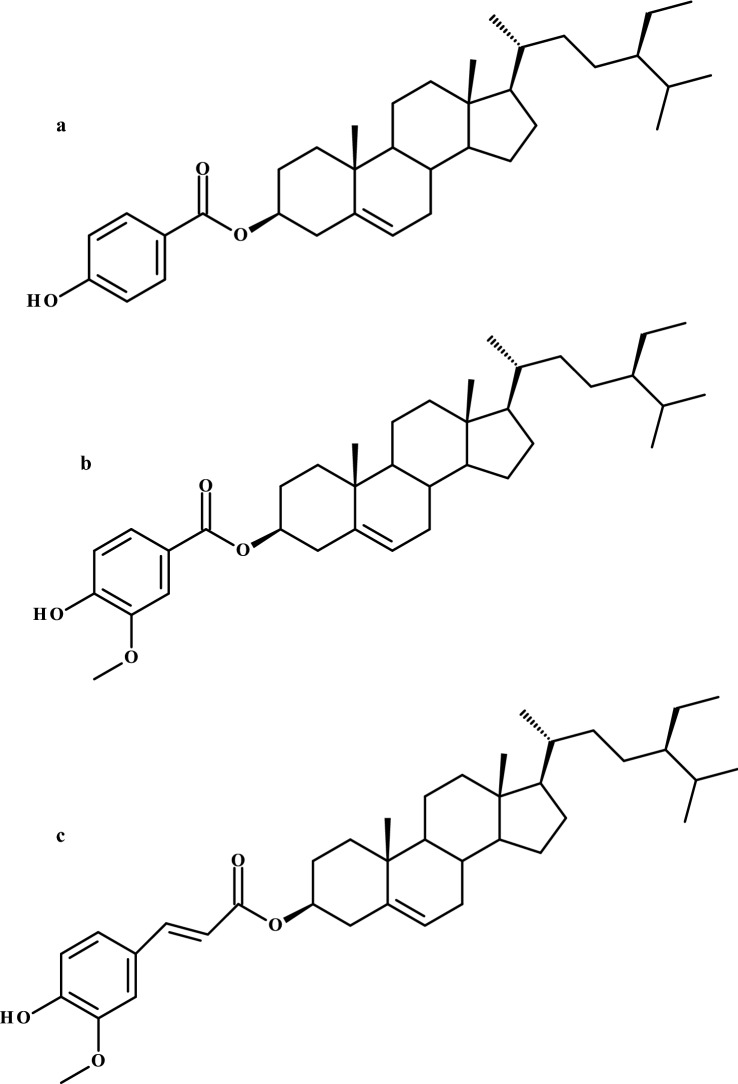
Chemical structures of **a** β-sitosteryl 4-hydroxybenzoate, **b** β-sitosteryl vanillate, and **c** β-sitosteryl ferulate

Lipoic acid and phytosterols play important roles in living organisms. Rideout et al. (2016) revealed that a combination of these compounds had better cholesterol-lowering properties than phytosterol or lipoic acid alone. Wang et al. (2018), for the first time proposed an enzymatic route for obtaining phytosteryl lipoate. The authors evaluated eight different parameters of the synthesis and the highest conversion rate achieved was 71.2%. Furthermore, the obtained ester was more soluble in rapeseed oil than phytosterols and enhanced oil stability similar to phytosteryl ferulate, the main ingredient of γ-oryzanol found in cereal grains with high antioxidant capacity (Wang et al. 2018). Moreover, 10 different phytosteryl phenolates were synthesized by Wang et al. (2015), using lipase from *C. rugosa* after process optimization in a hexane/2-butanone mixture (8:2 v/v) at 55 °C. The lipophilization of phenolic and arylalkanoic acids was achieved in a two-step chemoenzymatic synthesis. In the first stage, vinyl phenolates were obtained, and subsequently lipase-catalyzed transesterifications with phytosterols were performed. Three of them, namely phytosteryl 4-hydroxybenzoate (Fig. 7a), phytosteryl vanillate (Fig. 7b), and phytosteryl ferulate (Fig. 7c), showed potential uses as antioxidant agents and significantly inhibited the oxidation of linoleic acid (Wang et al. 2015).

Besides lipases, also other enzymes, such as laccases can be used in the enzymatic modification of chemical compounds. The aforementioned enzyme of *Pleurotus ostreatus* origin was used as a catalyst to modify the structure of chitosan, a linear polysaccharide composed of D-glucosamine and *N*-acetyl-D-glucosamine linked by β(1 → 4)-glycosidic bonds. Cinnamic acid and its derivatives: *p*-coumaric acid, ferulic acid, caffeic acid and chlorogenic acid were used as acyl donors. Intriguingly, resulted compounds exhibited varied antibacterial activity, and were defined as potential antimicrobial agents against potato wilt pathogen *Ralstonia solanacearum* GIM1.74, however, only the ester of chitosan and caffeic acid had sufficient antibacterial activity with IC_50_ of 0.23 mg/mL to suppress the growth of the mulberry wilt pathogen *R. solanacearum* RS − 5 (Yang et al. 2016).

Another example of the modification of polysaccharides can be found in the paper of Zhang et al. (2021). The authors investigated the lipase-catalyzed reaction between pectin and *o*-hydroxybenzoic, *m*-hydroxybenzoic or *p*-hydroxybenzoic acids in a two-phase system, and the emulsifying, antioxidant and antibacterial properties of the synthesized derivatives were evaluated. The introduction of phenolic acids to the pectin structure was dependent on the hydroxyl position in the phenolic ring. The substituent location was also crucial for emulsifying properties, increasing the inhibition ratio in the β-carotene bleaching assay and the antibacterial activity against *E. coli* and *S. aureus*. Modified pectins turned out to be interesting and perspective compounds, and especially the *p*-hydroxybenzoic derivative of pectin can find industrial application as a multitasking (antioxidant, antibacterial and emulsifying agent) compound in the near future (Zhang et al. 2021).

**Fig. 8:**
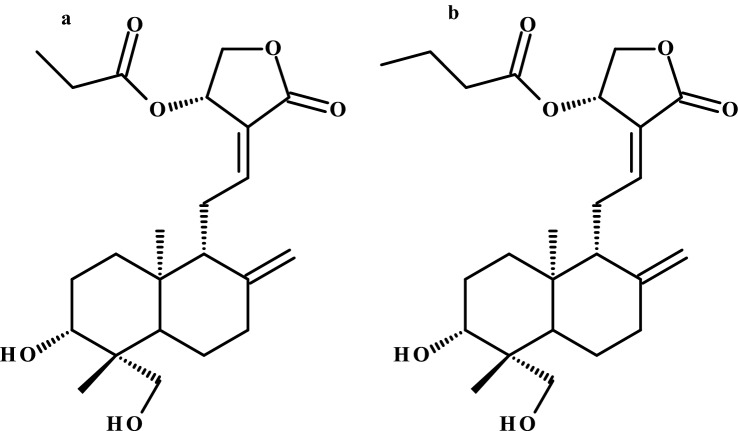
Chemical structures of **a** andrographolide-14-propionate and **b** andrographolide-14-butanoate

The production of low-molecular-weight esters as flavour compounds via lipase-catalyzed reactions is a useful green tool and has been known for many years (Vaidya et al. 2008). Terpenoids are another group of compounds that undergo enzymatic modifications, and terpenoids such as geraniol, citronellol or essential oils have also been used to obtain flavour compounds (Staudt et al. 2020). Patil et al. (2018) using Amano lipase AK (*Pseudomonas fluorescens*) synthesized five different compounds via transesterification of vinyl esters with andrographolide, a diterpene and major constituent of *Andrographis paniculata* with confirmed pharmacological activities. Andrographolide derivatives exhibited higher antibacterial activity than their precursor, and andrographolide-14-propionate (Fig. 8a) and andrographolide-14-butanoate (Fig. 8b) showed the highest antibacterial activity against *E. coli* and *S. aureus* with MIC ranging from 4–8 µg/mL, increased cell membrane permeability and low haemolysis activity (Patil et al. 2018).

## Conclusions and future perspectives

Due to the numerous beneficial health effects of the described compounds, enzymatic methods for the modification of chemical substances may contribute to the development of safe food, free from spoilage microorganisms, their metabolites and adverse biochemical changes. This review article shows that in many cases, the chemical compounds obtained via enzymatic modification have improved antioxidant and antimicrobial properties.

The tremendous application possibilities of multi-functioning compounds reveal that they could replace controversial food additives and can be an alternative to chemically derived food additives. Because of their multifaceted functions in the food product, they could reduce the use of other substances to maintain the same effect. Many of the reviewed compounds can also successfully be implemented in the cosmetics and pharmaceutical industry. The lipophilic esters of erythorbic acid and the derivatives of phenolic compounds have promising applications in the future. The trend of the biotransformation of multi-compound mixtures, such as polyphenol extracts, essential or microbial oils or polysaccharides and honeys, should be developed to allow for a significant reduction in the use of conventionally obtained food additives. The use of fully biotechnological processes for the modification of valuable compounds and their mixtures can further increase their beneficial activities and extend their use range in different industries.

## Supplementary Information

Below is the link to the electronic supplementary material.

Supplementary file1 (PDF 220 KB)
