# Supplementary material for: Recent advances in the enzymatic synthesis of lipophilic antioxidant and antimicrobial compounds
Source: World J Microbiol Biotechnol. 2021 Dec 7;38(1):11. doi: 10.1007/s11274-021-03200-5 (PMC8648661; doi:10.1007/s11274-021-03200-5)
Supplement: Supplementary file 1 — Supplementary file1 (PDF 220 KB) [file 11274_2021_3200_MOESM1_ESM.pdf]

**Table S1.** Antimicrobial activity of enzyme-synthesized esters.

| **Compound** | **Microbial strain** | **MIC (MMC)*** | **Inhibitory zone diameter** | **Reference** |
|---|---|---|---|---|
| hexyl ferulate | *L. monocytogenes* ATCC 19115 | 0.1 mM (0.2 mM) | - | Shi et al. 2019 |
| | *E. coli* ATCC 25922 | 1.6 mM (25.6 mM) | | Shi et al. 2018 |
| octyl 3-(4-hydroxyphenyl)propanoate | *L. monocytogenes* PCM 2191 | 0.0625 mM (0.25 mM) | - | Zieniuk et al. 2021a |
| erythorbyl myristate | *S. aureus* ATCC 12692 | 0.60 mM (0.73 mM) | - | Park et al. 2021 |
| | *S. aureus* ATCC 29213 | 0.60 mM (0.93 mM) | | |
| | *B. cereus* ATCC 10876 | 0.07 mM (0.08 mM) | | |
| | *B. cereus* ATCC 13061 | 0.06 mM (0.07 mM) | | |
| erythorbyl laurate | *S. aureus* ATCC 12692 | 0.88 mM (1.27 mM) | - | Park et al. 2018 |
| | *S. aureus* ATCC 29213 | 0.88 mM (1.27 mM) | | |
| | *S. aureus* ATCC 49444 | 0.48 mM (0.80 mM) | | |
| | *B. cereus* ATCC 13061 | 0.73 mM (0.90 mM) | | |
| | *B. cereus* ATCC 10876 | 0.65 mM (1.00 mM) | | |
| | *L. monocytogenes* ATCC 7644 | 0.58 mM (0.65 mM) | | |
| | *L. monocytogenes* ATCC 19115 | 0.53 mM (0.73 mM) | | |
| neokestose laurate | *S. aureus* CMCC 26071 | - | 12.43 ± 0.30 mm | Ning et al. 2017 |
| | *S. aureus* ATCC 25923 | | 13.45 ± 0.31 mm | |
| | *S. aureus* ATCC 6538 | | 13.01 ± 0.13 mm | |
| | *L. monocytogenes* 10403s | | 10.11 ± 0.28 mm | |
| | *L. monocytogenes* EGD | | 9.78 ± 0.22 mm | |
| | *L. monocytogenes* ScottA | | 10.32 ± 0.47 mm | |
| | *S. mutans* ATCC 35668 | | 12.23 ± 0.26 mm | |
| | *B. cereus* ATCC 11778 | | 12.89 ± 0.88 mm | |
| | *B. subtilis* ATCC 6051 | | 11.38 ± 0.42 mm | |
| | *E. coli* ATCC 44752 | | No Activity | |
| | *E. coli* ATCC 25922 | | No Activity | |
| | *E. coli* CICC 10372 | | No Activity | |
| | *P. aeruginosa* ATCC 9027 | | No Activity | |
| | *S. typhimurium* ATCC 50041 | | No Activity | |
| fructose laurate | *S. mutans* KCTC 3065 | < 0.39 mM | - | Lee and Kim 2016 |
| | *B. coagulans* KCTC 3625 | < 0.39 mM | | |
| | *G. stearothermophilus* KCTC 2107 | < 0.39 mM | | |

**Table S1.** Continued.

| | | | | |
|---|---|---|---|---|
| *C. echinulate* oil-glucose esters | *K. pneumoniae* ATCC 700603 | 25 µg/mL (100 µg/mL) | 10.5 ± 0.5 mm | El-Baz et al. 2021 |
| | *P. aeruginosa* ATCC 15442 | 41.7 µg/mL (100 µg/mL) | 6.8 ± 0.8 mm | |
| | *B. subtilis* ATCC 6633 | 16.7 µg/mL (50 µg/mL) | 14.1 ± 0.5 mm | |
| | *S. aureus* ATCC 25923 | 12.5 µg/mL (50 µg/mL) | 12.3 ± 0.2 mm | |
| *N. gaditana* oil-glucose esters | *K. pneumoniae* ATCC 700603 | 50 µg/mL (100 µg/mL) | 11.1 ± 0.4 mm | |
| | *P. aeruginosa* ATCC 15442 | 50 µg/mL (100 µg/mL) | 10.5 ± 0.5 mm | |
| | *B. subtilis* ATCC 6633 | 20.8 µg/mL (100 µg/mL) | 9.0 ± 0.0 mm | |
| | *S. aureus* ATCC 25923 | 50 µg/mL (83.3 µg/mL) | 8.6 ± 0.3 mm | |
| Olive oil-glucose esters | *K. pneumoniae* ATCC 700603 | 66.7 µg/mL (83.3 µg/mL) | 10.3 ± 0.6 mm | |
| | *P. aeruginosa* ATCC 15442 | 25 µg/mL (83.3 µg/mL) | 10.1 ± 0.4 mm | |
| | *B. subtilis* ATCC 6633 | 33.3 µg/mL (100 µg/mL) | 9.8 ± 0.7 mm | |
| | *S. aureus* ATCC 25923 | 83.3 µg/mL (100 µg/mL) | 12.0 ± 0.6 mm | |
| EPA Concentrate-glucose esters | *K. pneumoniae* ATCC 700603 | 33.3 µg/mL (100 µg/mL) | 14.2 ± 0.1 mm | |
| | *P. aeruginosa* ATCC 15442 | 20.8 µg/mL (41.7 µg/mL) | 13.2 ± 0.0 mm | |
| | *B. subtilis* ATCC 6633 | 10.4 µg/mL (41.7 µg/mL) | 17.0 ± 0.5 mm | |
| | *S. aureus* ATCC 25923 | 8.3 µg/mL (33.3 µg/mL) | 17.0 ± 0.2 mm | |
| andrographolide-14-propionate | *M. luteus* | 16 µg/mL | - | Patil et al. 2018 |
| | *S. aureus* | 4 µg/mL | | |
| | *P. fluorescens* | 32 µg/mL | | |
| | *E. coli* | 8 µg/mL | | |
| | *A. fumigatus* | > 128 µg/mL | | |
| | *R. oryzae* | > 128 µg/mL | | |
| | *C. albicans* | 16 µg/mL | | |
| andrographolide-14-butanoate | *M. luteus* | 32 µg/mL | | |
| | *S. aureus* | 8 µg/mL | | |
| | *P. fluorescens* | 16 µg/mL | | |
| | *E. coli* | 4 µg/mL | | |
| | *A. fumigatus* | > 128 µg/mL | | |
| | *R. oryzae* | > 128 µg/mL | | |
| | *C. albicans* | 64 µg/mL | | |
| sucrose monolaurate | *L. monocytogenes* ATCC 19114 | 2.5 mM (20 mM) | - | Shao et al. 2018 |
| | *B. subtilis* CMCC(B) 63501 | 2.5 mM (20 mM) | | |
| | *S. aureus* CMCC(B) 26003 | 10 mM (40 mM) | | |
| | *E. coli* CMCC(B) 44102 | > 80 mM | | |
| dilauryl azelate ester | *S. epidermidis* S273 | - | 9.0 ± 0.1 mm | Khairudin et al. 2018 |

* MIC – minimum inhibitory concentration; MMC – minimum microbicidal concentration
